# Supplementary material for: Painting with odors: How olfactory stimuli influence artistic expression, emotional response, visual perception, and object selection
Source: PLoS One. 2026 Mar 27;21(3):e0345917. doi: 10.1371/journal.pone.0345917 (PMC13029799; doi:10.1371/journal.pone.0345917)
Supplement: S1 Table — (DOCX) [file pone.0345917.s001.docx]

**S1 Table. Affective Dimensionality** and **Perceptual Valence and Complexity** Scores for Each Painting

| **participant** | **Affective Dimensionality scores (rose)** | **Affective Dimensionality scores (strawberry)** | **Perceptual Valence and Complexity scores (rose)** | **Perceptual Valence and Complexity scores (strawberry)** |
| --- | --- | --- | --- | --- |
| 1 | 15.28 | *18.72 | *22.12 | 16.48 |
| 2 | 14.45 | *19.17 | *19.52 | 15.95 |
| 3 | 15.48 | *18.98 | *19.5 | 16.35 |
| 4 | 15.17 | *18.62 | *20.15 | 16.73 |
| 5 | 14.88 | *19.12 | *21.8 | 15.55 |
| 6 | 15.03 | *18.12 | *18.95 | 16.8 |
| 7 | 14.95 | *17.55 | *20.33 | 15.52 |
| 8 | 15.63 | *18.58 | *19.57 | 17 |
| 9 | 15.95 | *18.45 | *20.1 | 16.27 |
| 10 | 15.75 | *17.98 | *20.55 | 16.4 |
| 11 | 14.77 | *15.5 | 19.68 | *20.53 |
| 12 | 15.57 | *16.13 | 19.3 | *21.33 |
| 13 | 16.03 | *18.42 | *21.73 | 16.78 |
| 14 | 14.7 | *19.48 | *19.92 | 16.33 |
| 15 | 15.03 | *16.17 | *20.95 | 19.47 |
| 16 | 15.58 | *15.53 | *18.82 | 19.98 |
| 17 | 15.5 | *18.23 | *20.28 | 16.73 |
| 189 | 14.68 | *17.48 | *18.65 | 16.3 |
| 19 | 15.07 | *18.07 | *19.87 | 16.22 |
| 20 | 16.13 | *18.6 | *21.78 | 15.68 |
| 21 | 15.45 | *18.15 | *22.38 | 14.25 |
| 22 | 15.05 | *18.88 | *19.47 | 16.57 |
| 23 | 15.95 | *19.07 | *21.48 | 16.42 |
| 24 | 15.45 | *18.93 | *19.93 | 16.03 |

Table S 1

Affective Tone and Preference Scores for Each PaintingUnder Rose and Strawberry Conditions. The score with * indicatesignificantly bigger than the paintings with another odor.
