# Supplementary material for: Painting with odors: How olfactory stimuli influence artistic expression, emotional response, visual perception, and object selection
Source: PLoS One. 2026 Mar 27;21(3):e0345917. doi: 10.1371/journal.pone.0345917 (PMC13029799; doi:10.1371/journal.pone.0345917)
Supplement: S2 Table — (DOCX) [file pone.0345917.s002.docx]

| Outcome | Contrast | Estimate_r_minus_s | SE | df | t | p_adj | Cohens_d | CI_d_lower | CI_d_upper |
| --- | --- | --- | --- | --- | --- | --- | --- | --- | --- |
| Affective Dimensionality | rose- strawberry | 2.56 | 0.295 | 48.2 | 8.666 | <.0001 | 1.12 | 0.865 | 1.38 |
| Perceptual Valence and Complexity | rose- strawberry | -1.81 | 0.199 | 49.1 | -9.095 | <.0001 | -1.06 | -1.3 | -0.829 |

**S2 Table. Pairwise contrasts (Bonferroni)**
