# Supplementary material for: Painting with odors: How olfactory stimuli influence artistic expression, emotional response, visual perception, and object selection
Source: PLoS One. 2026 Mar 27;21(3):e0345917. doi: 10.1371/journal.pone.0345917 (PMC13029799; doi:10.1371/journal.pone.0345917)
Supplement: S3 Table — (DOCX) [file pone.0345917.s003.docx]

**S3 Table.** Sensory-affective dimensions and hedonic olfactory valence scores for each evaluator under rose and strawberry conditions.

| **Evaluator** | **Sensory-Affective Dimensions**e **(Rose)** | **Sensory-Affective Dimensions**e **(strawberry)** | **Hedonic Olfactory Valence (strawberry)** | **Hedonic Olfactory Valence (Rose)** |
| --- | --- | --- | --- | --- |
| 1 | 17.00 | *20.00 | 4.00 | *12.00 |
| 2 | 15.00 | *17.00 | 5.00 | *13.00 |
| 3 | 15.00 | *17.00 | 3.00 | *11.00 |
| 4 | 12.00 | *16.00 | 4.00 | *8.00 |
| 5 | 13.00 | *21.00 | 6.00 | *13.00 |
| 6 | 13.00 | *18.00 | 7.00 | *10.00 |
| 7 | 8.00 | *21.00 | *8.00 | 7 |
| 8 | 10.00 | *16.00 | 5.00 | *6.00 |
| 9 | 11.00 | *16.00 | *8.00 | 4 |
| 10 | 10.00 | *16.00 | 6.00 | *7.00 |
| 11 | 14.00 | *21.00 | 4.00 | *6.00 |
| 12 | 14.00 | *23.00 | 6.00 | *7.00 |
| 13 | 16.00 | *21.00 | 4.00 | *7.00 |
| 14 | 9.00 | *16.00 | 4.00 | *6.00 |
| 15 | 12.00 | *17.00 | 5.00 | *7.00 |
| 16 | 10.00 | *15.00 | 5.00 | *6.00 |
| 17 | 8.00 | *20.00 | *10.00 | 4 |
| 18 | 6.00 | *20.00 | *5.00 | 4 |
| 19 | 8.00 | *19.00 | *7.00 | 5 |
| 20 | 11.00 | *19.00 | 10.00 | 10 |
| 21 | 12.00 | *21.00 | 6.00 | *10.00 |
| 22 | 16.00 | *17.00 | 6.00 | 6 |
| 23 | 14.00 | *16.00 | 5.00 | *8.00 |
| 24 | 15.00 | *23.00 | 5.00 | 5 |
| 25 | 14.00 | *21.00 | 6.00 | *12.00 |
| 26 | 9.00 | *15.00 | 4.00 | *8.00 |
| 27 | *19.00 | 16 | 4.00 | *11.00 |
| 28 | 14.00 | *21.00 | 5.00 | *7.00 |
| 29 | 15.00 | 15 | *6.00 | 4 |
| 30 | 15.00 | *18.00 | *5.00 | 4 |
| 31 | 14.00 | *18.00 | 5.00 | *6.00 |
| 32 | 12.00 | *19.00 | *8.00 | 7 |
| 33 | 14.00 | *18.00 | 6.00 | *7.00 |
| 34 | 13.00 | *17.00 | *7.00 | 5 |
| 35 | 11.00 | *18.00 | 8.00 | *13.00 |
| 36 | 8.00 | *14.00 | *10.00 | 9 |
| 37 | 12.00 | *18.00 | 8.00 | *10.00 |
| 38 | 12.00 | *20.00 | 7.00 | *10.00 |
| 39 | 12.00 | *21.00 | 7.00 | *12.00 |
| 40 | 12.00 | *20.00 | 9.00 | 9 |
| 41 | 8.00 | *20.00 | 7.00 | *14.00 |
| 42 | 9.00 | *23.00 | *11.00 | 10 |
| 43 | 9.00 | *20.00 | *17.00 | 14 |
| 44 | 7.00 | *16.00 | 11.00 | *17.00 |
| 45 | 9.00 | *19.00 | 12.00 | *13.00 |
| 46 | 10.00 | *16.00 | *15.00 | 10 |
| 47 | 11.00 | *13.00 | *19.00 | 18 |
| 48 | 8.00 | *19.00 | 10.00 | *16.00 |
| 49 | 12.00 | 820 | 14.00 | *15.00 |
| 50 | 10.00 | *17.00 | 12.00 | *13.00 |
| 51 | 13.00 | *19.00 | *14.00 | 12 |
| 52 | 12.00 | *16.00 | 11.00 | *16.00 |
| 53 | 9.00 | *17.00 | *11.00 | 9 |
| 54 | 11.00 | *14.00 | 10.00 | *12.00 |
| 55 | 16.00 | *22.00 | 8.00 | *11.00 |
| 56 | 14.00 | *22.00 | 8.00 | *11.00 |
| 57 | 12.00 | *17.00 | *10.00 | 7 |
| 58 | 13.00 | *18.00 | 12.00 | *13.00 |
| 59 | 12.00 | *18.00 | 11.00 | *14.00 |
| 60 | 17.00 | *18 | 6.00 | *10 |

presents the affective quality and preference scores given by evaluators for paintings created under Rose and Strawberry odor conditions. Scores marked with an * denote significantly higher ratings compared to the paintings associated with the other odors.
