# Supplementary material for: Painting with odors: How olfactory stimuli influence artistic expression, emotional response, visual perception, and object selection
Source: PLoS One. 2026 Mar 27;21(3):e0345917. doi: 10.1371/journal.pone.0345917 (PMC13029799; doi:10.1371/journal.pone.0345917)
Supplement: Attachment — Submitted filename: paper comments.docx [file pone.0345917.s004.docx]

The manuscript presents two well designed experiments that explore an understudied but increasingly relevant research area. The experiments are creative, and generally methodological sound.

However, the manuscript would benefit from clarifications in methodology, restructuring for clarity, statistical reporting improvements and an thorough proofread for the English language.

1. Why strawberry and rose, what is your rationale for these two odours?
2. For you first reference it would be beneficial to add the following reference to help support the claim;

Ward, R. J., Ashraf, M., Wuerger, S., & Marshall, A. (2023). Odors modulate color appearance. *Frontiers in psychology*, *14*, 1175703.

3. Line 51 – 54, would be worth mentioning there is more going on than just associative (i.e., hedonics), albeit associative is mostly like the strongest contributor in this case.
4. Investigation into the dynamics of visual attention reveals that background odors significantly affect visual behavior in tasks requiring rapid attentional deployment – reference?
5. It is hypothesized that exposure to strawberry and rose odors lead participants to

use distinct color palettes in their paintings and that paintings inspired by these odors are evaluated differently. – I think you need to strengthen this justification, one could argue that there could be a lot of overlap between these two colour pallets. i.e., green and red-pink, making them not distinct (maybe cultural impacted?). They are also both red, sweet, and pleasant smells.

6. You state that the odours were delivered with a diffuser. But at what concentration, was it consistent across participants?
7. Was there any ventilation in the room or attempt at odour contamination removal between participants?
8. Table 1, would be worth stating why Perfume and Berry are empty
9. What factor analysis algorithm did you use?
10. What criteria for factor retention did you use?
11. Table 2 might be better placed in the supplementary materials?
12. What factor rotation algorithm did you use?
13. Could you double check the GLM and AVOVA analysis the F-scores seem very high in places
14. Line 234 Bonferroni corrected? What were the specific comparisons and effect sizes, and exact p values? If your doing multiple tests where are the rest of the p-values?
15. Line 246 Principal component analysis?
16. For study 1, was the odour order presented randomly (i.e., first time it may or rose or strawberry or the control) or was it done consistently?
17. Can we see the first two loadings for the factor analysis in a graph?
18. Colour quantification more information is needed here, were images standardized for different lighting or scanned? Did the participants use identical paper and lighting setup (i.e., was the light controlled during the experiments). How was the number 25 choosen? Why k-means clustering, although a creative solution, were the colours converted to a perceptually modelled colour space or left as RGB?
19. Table 9, why is “No” now in the table, it doesn’t seem present in study 1, also what exactly does “No” mean in this context (no odour?)
20. Please be consistent when reporting you p-values (sometimes denoted as Sig), for example for Table 2, I assuming the * denote significance? What are their p values
21. I would go over the analysis again in its entirety, we need to know exactly what you did with the underlying data, we need to know all p-values
22. Inconsistent capitalisation in throughout the document
23. On lines 105-108 you mention a correlation might be present, could you perform this analysis or remove this statement if it isn’t applicable.
24. Table 8 can you perform statistical tests (possibly chi-squared tests) instead of just describing them?
25. Table 9 same issue above
26. Despite previous research illustrating the profound effects of odors on cognition and behavior [30–34], the results of this study expanded on existing findings by demonstrating both the nuanced and measurable impacts of olfactory stimuli on multisensory processing and artistic expression – please rephrase this sentence
27. This aligns with the findings from previous studies on olfactory and visual interactions[35,36]. They consistently showed that odors play a significant role in influencing artistic expression, guiding choices, and modulating emotional and visual perception. – I would expect more reference to be included in this statement, two seems very small given your claim here.
28. These results resonate with previous [37,38] studies on olfactory cues in consumer behavior, emphasizing the role of odors in shaping choices and attention. – again I would expect more references for this statement, a broad claim needs broad support.
29. Speaking of, you need to include more information about the odours themselves, how much mL of essential was used in the diffuser? How was the released controlled? Time? Intensity calibrated? Were the oils diluted or used neat? If diluted what was the ratio used, I assume this were mixed with water.
30. At the very end, can you place a very concise conclusion?
