# Supplementary material for: Painting with odors: How olfactory stimuli influence artistic expression, emotional response, visual perception, and object selection
Source: PLoS One. 2026 Mar 27;21(3):e0345917. doi: 10.1371/journal.pone.0345917 (PMC13029799; doi:10.1371/journal.pone.0345917)
Supplement: Attachment — Submitted filename: Response to reviweres.docx [file pone.0345917.s006.docx]

The manuscript presents two well designed experiments that explore an understudied but increasingly relevant research area. The experiments are creative, and generally methodological sound.

However, the manuscript would benefit from clarifications in methodology, restructuring for clarity, statistical reporting improvements and a thorough proofread for the English language.

Why strawberry and rose, what is your rationale for these two odours?

We thank the reviewer for raising this important point. We have now added a dedicated paragraph in the Introduction to clarify the rationale for selecting strawberry and rose odors. In brief, we chose these odors because (1) they are highly familiar and easily nameable in our international student population, (2) they are broadly matched in pleasantness and “sweet” hedonic tone, (3) they differ in semantic source category (edible fruit vs. non-edible flower/cosmetic product), and (4) there is existing evidence that fruity and floral odors show reliable odor–color correspondences. This combination allowed us to examine how odors with similar valence, but different ecological and semantic properties, are mapped onto colors and expressed in paintings (lines 105-116)

**Revised text in the manuscript (Introduction):**

“Strawberry and rose odors were selected because they provide a theoretically controlled contrast along semantic and ecological dimensions while remaining comparable in hedonic valence. Both odors are highly familiar and easily identifiable across cultures, making them suitable for use with an international student population (R. S. Herz, 2004; Levitan et al., 2014). Although strawberry and rose odors are both typically perceived as pleasant and are often associated with red or pink hues, converging evidence indicates that odor–color associations are not determined by hedonic valence alone but are strongly influenced by semantic category and object-based knowledge (Deroy et al., 2013; Luisa Dematte, 2006). Strawberry represents a sweet, fruity, and edible odor that is closely linked to gustatory experience and food-related representations, whereas rose is a floral odor associated with botanical environments and culturally embedded symbolic meanings such as elegance or romance (Spence, 2020).”

For your first reference it would be beneficial to add the following reference to help support the claim.

Ward, R. J., Ashraf, M., Wuerger, S., & Marshall, A. (2023). Odors modulate color appearance. *Frontiers in psychology*, *14*, 1175703.

We appreciate this suggestion. We have added Ward et al. (2023) as a reference in our Introduction to reinforce our statement about odors influencing color perception. The revised manuscript now includes Ward et al. (2023) as a supporting reference for the crossmodal influence of odors on color processing.

**Revised text in the manuscript (Introduction):**

The attributes of odor stimuli contribute to cross-modal correspondences between odors and visual characteristics, such as color dimensions (Ward et al., 2023).

Line 51 – 54, would be worth mentioning there is more going on than just associative (i.e., hedonics), albeit associative is mostly like the strongest contributor in this case.

Thank you for this helpful suggestion. We agree that odor–color correspondences cannot be explained solely by associative learning.

Accordingly, we revised Lines 72–88 to explicitly acknowledge the contribution of hedonic and affective factors alongside learned associations. The revised text now clarifies that while associative learning is likely the dominant mechanism, hedonic valence and emotional responses to odors also play a substantial role in shaping odor–color mapping (Lines 72-88).

**Revised text in the manuscript (Introduction ):**

“Odor–color correspondence should be interpreted within the broader framework of odor association learning rather than as an isolated crossmodal mapping. Evidence from taste–odor learning demonstrates that odors are preferentially associated with the **emotional (hedonic) aspects** of gustatory information, rather than with its qualitative sensory features. Using higher-order conditioning paradigms, Onuma and Sakai showed that odors acquire associative value primarily through emotional valence, with second-order conditioning emerging only when affective information is available, whereas associations based on sensory quality alone are comparatively weak (Onuma et al., 2016). Complementary human studies further indicate that visual information systematically modulates odor perception via top-down cognitive processes: visual cues such as colors or images evoke learned expectations that influence perceived odor intensity, preference, and identification (Sakai, 2005) These findings suggest that odor–visual correspondences, including odor–color associations, arise from learned multimodal associations grounded in hedonic evaluation and cognitive expectation. From this perspective, color functions as a visual manifestation of affective and semantic associations linked to odors, rather than as a direct sensory counterpart, providing a theoretical basis for understanding odor-induced modulation of visual expression.”

Investigation into the dynamics of visual attention reveals that background odors significantly affect visual behavior in tasks requiring rapid attentional deployment – reference?

Thank you for this comment. We have revised this statement and added appropriate references in the **Introduction** to explicitly support the claim that background odors influence visual attention. Specifically, prior studies have demonstrated that ambient odors modulate rapid visual attentional processes, including reflexive orienting, visual search, and attentional capture. These references have now been added to **lines 47–72** of the revised manuscript.

**Revised text in the manuscript (Introduction):**

“Recent studies indicate that ambient olfactory cues can bias rapid visual attention. For example, in an involuntary “attentional capture” paradigm, Michael et al. (2003) found that background odors modulated the reflexive orienting to sudden luminance onsets (Michael et al., 2003). In a follow-up task, the same group (Michael et al., 2005) showed that a trigeminal (irritant) odor (allyl isothiocyanate) increased both the magnitude and duration of attentional capture, whereas a pleasant rose‐like odor (phenyl ethyl alcohol) effectively abolished the capture effect and generally slowed processing (Michael et al., 2005). Parallel findings emerge in visual search and scanning tasks: for instance, Seo et al. (2010) used eye-tracking to show that smelling a congruent odor caused observers to make more and longer fixations on matching objects than in an odorless condition (Seo et al., 2010), and Seigneuric et al. (2010) similarly found that congruent ambient scents led participants to locate odor-associated targets more quickly during free viewing (Seigneuric et al., 2010). Chen et al. (2013) extended this by demonstrating in dot-probe and search paradigms that a smell reflexively draws the attentional “spotlight” to a semantically matching image, facilitating detection even against competing distractors (Chen et al., 2013). Neurophysiological evidence also supports odor-driven attentional shifts: Zhang et al. (2021) recorded EEG during an Attention Network Test and found that an unpleasant odor elicited larger early N1/N2 components to alerting cues and faster behavioral alerting responses, suggesting heightened preparatory attention for negative odors (Zhang et al., 2021). Castellotti et al. (2025) reported that ambient fruit odors congruent with the search target improved visual search performance, yielding higher accuracy and faster responses for matching targets than for incongruent or no-odor trials (Castellotti et al., 2025). These behavioral and neurocognitive studies suggest that background odors can bias rapid visual attentional deployment—via affective congruency and arousal mechanisms—altering eye movements, reaction times, and early neural processing of visual cues.”

It is hypothesized that exposure to strawberry and rose odors lead participants to

use distinct color palettes in their paintings and that paintings inspired by these odors are evaluated differently. – I think you need to strengthen this justification, one could argue that there could be a lot of overlap between these two-colour pallets. i.e., green and red, pink, making them not distinct (maybe cultural impacted?). They are also both red, sweet, and pleasant smells.

We understand the concern and have strengthened the justification for this hypothesis. In the revised Introduction, we now elaborate that although both strawberry and rose are pleasant and share some red-associated imagery, they also have distinct qualities that could lead to different artistic outcomes. Strawberry (a sweet, fruity odor) might evoke imagery of fruits, whereas rose (a floral odor) might evoke nature or botanical imagery. We acknowledge that some overlap in color choices could occur, but we argue that the different semantic and emotional associations of a fruit versus a flower scent provide a basis to expect differences in color palettes and art evaluations. This clarification has been added to make our rationale more convincing (Lines 109-116).

**Revised text in the manuscript: (Introduction)**

“Although strawberry and rose odors are both typically perceived as pleasant and are often associated with red or pink hues, converging evidence indicates that odor–color associations are not determined by hedonic valence alone but are strongly influenced by semantic category and object-based knowledge (Deroy et al., 2013; Luisa Dematte, 2006). Strawberry represents a sweet, fruity, and edible odor that is closely linked to gustatory experience and food-related representations, whereas rose is a floral odor associated with botanical environments and culturally embedded symbolic meanings such as elegance or romance (Spence, 2020).”

You state that the odours were delivered with a diffuser. But at what concentration, was it consistent across participants?

We thank the reviewer for requesting further clarification regarding the odor diffusion procedure. In the revised manuscript, we have described the method in a device-independent manner. The odor stimuli consisted of **vaporized aroma (essential oil)** applied to **absorbent cotton** and presented to participants at a fixed distance, allowing controlled olfactory stimulation without direct contact. For each experimental session, a fixed amount of essential oil (**10 drops; approximately 0.5 mL**) was applied to the cotton and used **neat (undiluted)**. Odor delivery was controlled procedurally by maintaining identical preparation methods, presentation distance, room size, and exposure duration across all participants within each odor condition. A fixed pre-exposure period was implemented to allow the odor to stabilize before participant entry, and presentation continued throughout the task.

The exact airborne concentration of the odor was not chemically quantified. However, consistency of odor exposure was ensured by standardized procedures and supported behaviorally by the high agreement in odor identification and labeling across participants, indicating comparable perceived odor intensity within each condition (Lines 183-191).

**Revised text in the manuscript: (Procedure and design)**

The odor stimuli were delivered as vaporized aroma by applying essential oil to absorbent cotton and presenting it to participants at a fixed distance, allowing controlled olfactory stimulation without direct contact. For each experimental session, a fixed volume of undiluted essential oil (10 drops; approximately 0.5 mL) was applied to the cotton to ensure consistency across trials. Diffusion settings, room size, and exposure duration were kept constant across all participants within each odor condition. A fixed pre-diffusion period was used prior to participant entry, and diffusion continued throughout the experimental task. Airborne odor concentration and intensity were not chemically calibrated.

Was there any ventilation in the room or attempt at odour contamination removal between participants?

Yes, we took measures to prevent odor carryover between participants. We have now described in the Methods that after each participant’s session, the room was ventilated (doors/windows opened and an interval allowed for air circulation) and the diffuser was turned off well in advance of the next session. To prevent odor carryover, sessions involving different odor conditions were separated by a **minimum interval of 48 hours**, and the experimenter confirmed that no residual odor remained before the next session. These steps ensured that the baseline odor condition was reset and that no residual scent from a previous session would affect the next participant (Lines 193-196).

**Revised text in the manuscript: (Procedure and design)**

“A minimum interval of 48 hours was maintained between sessions to minimize potential carryover effects. Before the start of each subsequent session, the experimenter confirmed that no residual odor from the previous condition remained in the experimental room. We opened windows and used an electric fan to dissipate any remaining odor, ensuring that each participant started in a neutral olfactory environment. The order of odor conditions in Study 1 was counterbalanced across participants. Each painter experienced the two odor conditions (strawberry, rose) in random order.”

Table 1 would be worth stating why Perfume and Berry are empty.

Thank you for this suggestion. In Study 1, participants did not report perceiving or labeling any odors as Berry or Perfume. As a result, no responses were recorded in these categories, which led to the empty cells in Table 1. To avoid potential confusion, we have revised Table 1 by **removing the** Berry **and** Perfume **categories from the Study 1 table**, so that it now includes only odor labels that were actually reported by participants in that study.

What factor analysis algorithm did you use?

Exploratory factor analysis was conducted using **principal axis factoring (PAF)** with varimax rotation. This information has been added to the **Methods (Analysis section)** of the revised manuscript (Lines 232-236).

**Revised text in the manuscript: (Analysis section)**

“An exploratory factor analysis (EFA) was conducted on the semantic differential ratings to identify underlying perceptual dimensions. Prior to factor extraction, sampling adequacy was confirmed using the Kaiser–Meyer–Olkin (KMO) measure and Bartlett’s test of sphericity. Factors were extracted using principal axis factoring (PAF) with varimax rotation.”

What criteria for factor retention did you use?

Thank you for this important question. In the revised manuscript, we explicitly state that we used three complementary criteria for factor retention:
(1) Kaiser’s criterion (eigenvalues > 1.0),
(2) visual inspection of the scree plot, and
(3) parallel analysis.
All procedures converged on a two-component solution, which we now report in the Methods and Results sections (Lines 273-276)

**Revised text in the manuscript (Results section):**

“An exploratory factor analysis of the eight semantic rating scales revealed a two-factor solution. Based on eigenvalues greater than 1 (λ₁ = 4.32; λ₂ = 1.07), the two factors together explained 67.3% of the total variance.”

Table 2 might be better placed in the supplementary materials.

We agree that this table can be moved out of the main text. In the revision, we have relocated Table 2 to the Supplementary Materials. This keeps the flow of the main manuscript concise while still providing the data for readers who are interested.

What factor rotation algorithm did you use?

We thank the reviewer for requesting clarification. We applied **varimax rotation** to the extracted factors. This information has been added to the **Methods (Analysis section)** of the revised manuscript (Lines 235-236).

**Revised text in the manuscript (Methods – Analysis section):**

“Factors were extracted using principal axis factoring (PAF) with varimax rotation.”

Could you double-check the GLM and AVOVA analysis the F-scores seem very high in places?

Thank you for raising this point. The statistical analyses were carefully re-examined. The originally reported ANOVA results were descriptive and did not fully account for the non-independence arising from repeated ratings by evaluators and repeated paintings by painters. To address this concern, the primary analyses were re-conducted using linear mixed-effects models with Odor specified as a fixed effect and Painter and Evaluator specified as random intercepts.

Using this approach, the effect of odor remained highly significant for both outcome measures (Table 4). The observed F-values reflect large and reliable effects, as supported by the corresponding effect sizes (partial η² = 0.62 for Affective Dimensionality and partial η² = 0.64 for Perceptual Valence and Complexity). The manuscript has been revised to clarify that statistical inference is based on mixed-effects models, with ANOVAs retained only as descriptive checks (Lines 238–245 and 306–310).

**Revised text in the manuscript (Methods – Analysis section):**

“We analyzed the data using linear mixed-effects models (LMMs) with Odor as a fixed effect and random intercepts for Painter and Evaluator to account for repeated measurements. Models were fitted using maximum likelihood (lmerTest), and degrees of freedom were estimated using the Kenward–Roger/Satterthwaite approximation.”

Revised text in the manuscript (Results section):

“As reported in Table 4, odor had a significant main effect on both outcome measures. For affective Dimensionality, a significant effect of odor was observed, F(1, 47.15) = 76.864, p = 1.82 × 10⁻¹¹, partial η² = 0.62. Similarly, for perceptual Valence and Complexity, the effect of odor was also significant, F(1, 47.78) = 85.477, p = 3.24 × 10⁻¹², partial η² = 0.64.”

Line 234 Bonferroni corrected. What were the specific comparisons and effect sizes, and exact p values? If you’re doing multiple tests where are the rest of the p-values?

Thank you for this important clarification request. All post hoc comparisons were conducted using estimated marginal means derived from the mixed-effects models, with Bonferroni correction applied across outcomes. The specific comparison of interest was between the rose and strawberry odor conditions.

In addition, the complete R analysis script has been included as Supplementary Material to facilitate reproducibility. We also provide complete tables of estimated marginal means and contrasts (including adjusted p-values) in the Supplementary Materials (Lines 319-326).

**Revised text in the manuscript (Results section):**

“Planned pairwise comparisons between odor conditions were conducted using estimated marginal means with Bonferroni correction (S2 Table, Pairwise contrasts). For affective Dimensionality, the contrast between rose and strawberry was significant (p < .0001, Cohen’s d = 1.12, 95% CI [0.865, 1.38]). For perceptual Valence and Complexity, the contrast was also significant in the opposite direction (p < .0001, Cohen’s d = −1.06, 95% CI [−1.30, −0.829]).”

Line 246 Principal component analysis?

We thank the reviewer for requesting further statistical analyses. To address this, we conducted a two-factor analysis examining odor (rose vs. strawberry) and evaluative dimension (affective evaluative vs. perceptual–sensory). Because the data involved repeated measures and some composite scores contained missing values, we employed linear mixed-effects modeling with participant as a random intercept. This analysis revealed a significant odor × dimension interaction, indicating that odor effects differed across evaluative dimensions. Bonferroni-corrected post-hoc tests further clarified the direction of these effects. These analyses have now been added to the Results section (Lines 333-339), (Lines 336-370)

**Revised text in the manuscript (Results – Odor rating phase):**

“An exploratory factor analysis (EFA) was conducted on the semantic differential ratings to examine the underlying structure of odor evaluations. Sampling adequacy was confirmed (KMO = 0.82), and Bartlett’s test of sphericity indicated that the correlation matrix was suitable for factor analysis…”

**Revised text in the manuscript (Results – Interaction analysis):**

“A linear mixed-effects model was fitted with odor (rose vs. strawberry), dimension (affective evaluative vs. perceptual–sensory), and their interaction as fixed effects, and participant as a random intercept. The analysis revealed a significant odor × dimension interaction, F (1, 236) = 29.06, p < .001, indicating overall higher ratings in the perceptual–sensory dimension.”

For study 1, was the odour order presented randomly (i.e., first time it may be rose or strawberry or the control) or was it done consistently?

In Study 1, participants experienced all odor conditions in a within-subject design, and we randomized the order of odor presentation for each participant. We have now clarified in the Methods that the sequence of odor conditions was counterbalanced across participants (some started with the strawberry condition, others with rose, in a varied order). This randomization ensured that no order effects biased the results (Lines 192-196).

**Revised text in the manuscript (Methods – Procedure and design, Study 1):**

“A minimum interval of 48 hours was maintained between sessions to minimize potential carryover effects. Before the start of each subsequent session, the experimenter confirmed that no residual odor from the previous condition remained in the experimental room. After completing each painting, they were asked to smell the odor again and report the colors they imagined from the odor and the name of the odor. To avoid cross-participant odor contamination, the room was thoroughly ventilated for 10 minutes between sessions. We opened windows and used an electric fan to dissipate any remaining odor, ensuring that each participant started in a neutral olfactory environment. The order of odor conditions in Study 1 was counterbalanced across participants. Each painter experienced the two odor conditions (strawberry, rose) in random order.”

Can we see the first two loadings for the factor analysis in a graph?

We appreciate this excellent suggestion. In the revised manuscript, we now include a **two-dimensional loading plot**illustrating the loadings of each semantic rating scale on the first two extracted factors. The figure is provided as Figure **6**.

**Figure 6. Varimax-rotated factor loading plot for the odor-rating exploratory factor analysis (principal axis factoring).**

Colour quantification more information is needed here, were images standardized for different lighting or scanned? Did the participants use identical paper and lighting setup (i.e., was the light controlled during the experiments). How was the number 25 choosen? Why k-means clustering, although a creative solution, were the colours converted to a perceptually modelled colour space or left as RGB?

We thank the reviewer for requesting additional clarification regarding color quantification (Lines 220-231).

**Revised text in the manuscript (Methods – Analysis section):**

“Color information was extracted from each image by converting pixel values from RGB to the CIELAB color space using Python, which provides a perceptually more uniform representation of color differences than device-dependent RGB. Dominant colors were identified using k-means clustering, a widely used color quantization method in vision science and computational aesthetics that summarizes an image’s color composition in a data-driven manner (Pavan Kumar et al., 2020; Westland et al., 2024). The number of clusters was fixed at 25 for all paintings, corresponding to a 5 × 5 color palette. This choice reflects a balance between capturing fine-grained color variation and maintaining consistency across images, and is consistent with prior work using medium-sized palettes to represent artwork color composition (Westland et al., 2024). The resulting color palettes provided a standardized representation of color usage across experimental conditions and were used for subsequent statistical analyses.”

Table 9, why is “No” now in the table? It doesn’t seem present in study 1, what exactly does “No” mean in this context (no odour?)

Thank you for this important point. In Study 2, a no-odor control condition was included in which no scent was intentionally diffused in the room. The category “No” in Table 9 refers to participants who reported not sensing any odor during this control condition. This category does not appear in Study 1 because that study did not include a no-odor control condition. The table caption and accompanying text have been revised to clarify this distinction and to explicitly define the meaning of “No” as the absence of perceived odor (Lines 540-542).

**Revised text in the manuscript (Results – Study 2):**

“In the no odor control condition, participants reported not sensing any smell; these responses are labeled as ‘No’ in Table 10.”

Please be consistent when reporting you p-values (sometimes denoted as Sig), for example for Table 2, I assuming the * denote significance? What are their p values?

We have standardized the reporting of p-values throughout the manuscript. We removed inconsistent notations like “Sig” and now report actual p-values (or significance levels) uniformly. For Table 2 and other tables, we have added footnotes to explain any asterisks and provided the exact p-values associated with them. This ensures consistency and clarity in our statistical reporting.

I would go over the analysis again in its entirety, we need to know exactly what you did with the underlying data, we need to know all p-values.

We have taken this advice and thoroughly reviewed the analysis section. We ensured that every analysis is described with sufficient detail and that all statistical tests and results are fully reported. In the revised manuscript, we now explicitly describe each data processing step and analysis performed, and we report all p-values (along with test statistics and effect sizes) for every result discussed. This provides complete transparency about our methods and findings.

Inconsistent capitalisation in throughout the document

Thank you for pointing this out. We have carefully reviewed the manuscript and standardized capitalization throughout, ensuring consistent use of capitalization for headings, figure and table titles, variable names, and technical terms in accordance with the journal’s style guidelines.

On lines 105-108 you mention a correlation might be present, could you perform this analysis or remove this statement if it isn’t applicable.

Thank you for this helpful suggestion. To avoid overinterpretation, the sentence referring to a potential correlation has been removed from the manuscript.

Table 8 can you perform statistical tests (possibly chi-squared tests) instead of just describing them?

We thank the reviewer for this suggestion. We have now conducted chi-square tests of independence on the raw choice data from Study 2. The overall test revealed a significant association between odor condition and choice (χ² = 52.37, Monte Carlo p < .001), with a large effect size (Cramér’s V = 0.62). Follow-up pairwise comparisons using Monte Carlo chi-square tests with Bonferroni correction showed that all odor conditions differed significantly from each other (adjusted ps < .001). These analyses have been added to the Results section (Lines 522-526).

**Revised text in the manuscript (Results – Study 2)**

“A chi-square test of independence using Monte Carlo simulation revealed a significant association between odor condition and choice (χ² = 52.37, p < .001), with a large effect size (Cramér’s V = 0.62). Follow-up pairwise comparisons (Bonferroni-corrected) indicated that all odor conditions differed significantly from each other.”

Table 9 same issue above

Thank you for this comment. We have now conducted chi-square tests of independence for the data presented in Table 9. Because participants in the no-odor condition did not report odor names, statistical analysis was restricted to the strawberry and rose odor conditions. A chi-square test using Monte Carlo simulation revealed a significant association between odor condition and reported odor name, and these results have been added to the Results section (Lines 545-550).

**Revised text in the manuscript (Results – Study 2)**

“For reported odor names, statistical analysis was restricted to the strawberry and rose odor conditions, as participants in the no-odor condition did not report odor labels. A chi-square test of independence using Monte Carlo simulation revealed a significant association between odor condition and reported odor name (χ² = 40.00, p < .001), with a very large effect size (Cramér’s V = 0.96), indicating distinct odor identification patterns across conditions.”

Despite previous research illustrating the profound effects of odors on cognition and behavior [30–34], the results of this study expanded on existing findings by demonstrating both the nuanced and measurable impacts of olfactory stimuli on multisensory processing and artistic expression – please rephrase this sentence.

Thank you for this comment. We have rephrased the sentence in the Discussion to improve clarity and readability. The revised version clearly distinguishes prior research on the effects of odors on cognition and behavior from the contribution of the present study, which shows measurable effects of olfactory stimuli on multisensory processing and aspects of artistic expression (Lines 578-584).

**Revised text in the manuscript (General Discussion):**

“Across two studies, this research explored the interplay between olfactory and visual perception. It revealed consistent evidence of odors' influence on creative expression and choice preference. This interpretation is consistent with a substantial body of research on olfactory–visual and crossmodal interactions, demonstrating that sensory information from one modality can systematically shape perception, evaluation, and expressive outcomes in another modality rather than serving merely as contextual background (Luisa Dematte, 2006; Seo et al., 2010; Ward et al., 2023).”

This aligns with the findings from previous studies on olfactory and visual interactions [35,36]. They consistently showed that odors play a significant role in influencing artistic expression, guiding choices, and modulating emotional and visual perception. – I would expect more reference to be included in this statement, two seems very small given your claim here.

We thank the reviewer for this helpful suggestion. We agree that the original statement required a broader and more representative citation base. In the revised manuscript, we have substantially expanded the number of references supporting this claim to include additional empirical and theoretical studies on olfactory–visual interactions, emotional modulation, attentional bias, choice behavior, and crossmodal perception. Specifically, we now cite work demonstrating that odors bias visual attention and perception (Demattè et al., 2006; Seo et al., 2010; Zhou et al., 2012), modulate emotional evaluation of visual stimuli (Demattè et al., 2007; Syrjänen et al., 2019), influence preference formation and choice behavior (Li et al., 2007; Herz, 2009), and shape creative and expressive processes (Knasko, 1992). In addition, we have incorporated a broader theoretical framework on crossmodal interactions to contextualize these findings (Ward, 2013; Ward et al., 2006) (Lines 584-592).

**Revised text in the manuscript (General Discussion):**

“Previous studies have shown that odors can bias visual attention and perceptual (Seo et al., 2010; Zhou et al., 2012), alter aesthetic and evaluative judgments of visual stimuli such as faces and artworks (Dematte et al., 2008), and modulate emotional responses to visual scenes (Syrjänen et al., 2019). More broadly, research on crossmodal perception and synesthetic-like correspondences indicates that interactions between sensory modalities can influence higher-level cognitive processes, including preference formation, creative expression, and decision-making (Banissy et al., 2014; Rachel S. Herz, 2009; Knasko, 1992; WARD et al., 2006). Together, this literature supports the view that olfaction plays an active and integrative role in multisensory processing, shaping how visual information is perceived, emotionally interpreted, and translated into artistic and expressive decisions.”

These results resonate with previous [37,38] studies on olfactory cues in consumer behavior, emphasizing the role of odors in shaping choices and attention. – again, I would expect more references for this statement, a broad claim needs broad support.

We thank the reviewer for this valuable comment and agree that the original statement required broader empirical support. In the revised manuscript, we have expanded the reference base to include a wider range of foundational and contemporary studies from consumer psychology and sensory marketing. These studies demonstrate that olfactory cues influence attention and evaluative processing (Spangenberg et al., 1996; Morrin & Ratneshwar, 2000), shape preference formation and affective responses (Bone & Ellen, 1999; Herz & Engen, 1996), and modulate choice-related and approach behaviors such as time spent, impulse buying, and revisit intentions in both laboratory and real-world settings (Mattila & Wirtz, 2001; Li et al., 2007; Krishna, 2012) (lines 617-621).

**Revised text in the manuscript (General Discussion):**

“These results resonate with a substantial body of consumer research showing that ambient olfactory cues can influence attention and evaluations/preferences, and can shift approach-related behaviors (e.g., time spent, impulse buying, revisit intentions) in both controlled and real-world retail settings. (Fitzgerald Bone et al., 1999; Rachel S. Herz et al., 1996; Krishna, 2012; Leenders et al., 2019; Li et al., 2007; Morrin et al., 2000; Spangenberg et al., 1996).”

Speaking of you need to include more information about the odours themselves, how much mL of essential was used in the diffuser? How was the released controlled? Time? Intensity calibrated? Were the oils diluted or used neat? If diluted what was the ratio used, I assume this were mixed with water.

We thank the reviewer for requesting additional methodological detail regarding odor delivery. In the revised manuscript, we have clarified the odor diffusion procedure in a device-independent manner.

The odor stimuli consisted of **vaporized essential oil presented via absorbent cotton,** which allowed controlled olfactory stimulation without direct contact. For each experimental session, a fixed amount of essential oil **(10 drops; approximately 0.5 mL)** was applied to the cotton and used **neat (undiluted).**

Odor release was controlled procedurally rather than chemically. The same preparation method, room size, presentation distance, and exposure duration were used for all participants within each odor condition. A fixed pre-exposure period was implemented to allow the odor to stabilize in the room before participant entry, and odor presentation was maintained throughout the task. These clarifications have now been added to the Methods section (Lines 183-191).

**Revised text in the manuscript (Methods – Procedure and design, Study 1):**

“The odor stimuli were delivered as vaporized aroma by applying essential oil to absorbent cotton and presenting it to participants at a fixed distance, allowing controlled olfactory stimulation without direct contact. For each experimental session, a fixed volume of undiluted essential oil (10 drops; approximately 0.5 mL) was applied to the cotton to ensure consistency across trials. Diffusion settings, room size, and exposure duration were kept constant across all participants within each odor condition. A fixed pre-diffusion period was used prior to participant entry, and diffusion continued throughout the experimental task. Airborne odor concentration and intensity were not chemically calibrated.”

At the very end, can you place a very concise conclusion?

We thank the reviewer for this helpful suggestion. In response, we have added a concise concluding paragraph at the very end of the manuscript. This conclusion briefly synthesizes the main findings from both studies, emphasizing the consistent influence of olfactory stimuli on color perception, artistic expression, and object selection, as well as the broader implications for multisensory processing. This addition improves clarity and provides a clear takeaway message for readers (Lines 680-685).

**Revised final paragraph of the manuscript:**

“These studies confirm robust cross-modal correspondences: strawberry and rose odors evoked warm (yellow/orange) versus cool (blue/green) color palettes, biasing participants’ color choices in their paintings and subtly influencing their object selections. Such results underscore olfaction’s profound multisensory influence on perception and behavior, highlighting that ambient odors can implicitly guide visual perception and creative decision-making.”
