# Supplementary material for: Painting with odors: How olfactory stimuli influence artistic expression, emotional response, visual perception, and object selection
Source: PLoS One. 2026 Mar 27;21(3):e0345917. doi: 10.1371/journal.pone.0345917 (PMC13029799; doi:10.1371/journal.pone.0345917)
Supplement: Attachment — Submitted filename: Response to Academic Editor.docx [file pone.0345917.s007.docx]

Response to Academic Editor

We sincerely thank the Academic Editor for the careful review and for the helpful suggestions to improve methodological clarity and transparency. We have addressed each point below and revised the manuscript accordingly.

1. Clarification of odor delivery procedure

Comment:
We thank the Academic Editor for this important request for clarification. We have revised the Methods section to make explicit that the absorbent cotton and diffuser were not separate delivery methods but components of a single integrated odor-delivery system.

Specifically, essential oil was first applied directly to the internal absorbent cotton pad (10 drops; approximately 0.5 mL), and the cotton pad was then placed inside the Aroma Breeze Nova Diffuser. The diffuser subsequently dispersed the odor from this cotton carrier. Thus, the cotton served as the odor substrate, and the diffuser functioned as the dispersion mechanism.

We have further clarified:

- The applied volume (10 drops; ~0.5 mL per session)
- The exposure mode in Study 1 (brief, near-participant inhalation without continuous ambient diffusion)
- The exposure mode in Study 2 (continuous room diffusion using 2 symmetrically positioned diffusers)
- The timing and duration of odor exposure in both studies

These clarifications have been incorporated into the revised Methods section to improve procedural transparency and reproducibility.

Lines 158-160, 178-185, 473-475, 488-496 :

Study 1: This diffuser uses an internal absorbent cotton pad as the odor carrier; essential oil was applied directly to this cotton, which served as the sole source of odor delivery. The odor stimuli were delivered as a vaporized aroma using the Aroma Breeze Nova Diffuser, in which essential oil was applied to an internal absorbent cotton pad that served as the odor source. For each experimental session, a fixed volume of undiluted essential oil (10 drops; approximately 0.5 mL) was applied to the cotton to ensure consistency across trials. In Study 1, odor exposure was delivered as brief, direct inhalation rather than continuous room odorization. Participants were seated approximately 20–30 cm from the diffuser and instructed to inhale the odor for approximately 10 seconds prior to beginning the painting task.

Study 2: The diffuser operates using an internal absorbent cotton pad to which essential oil was applied; this cotton pad served as the sole odor source throughout the experiment. In Study 2, to promote homogeneous odor distribution, two identical Aroma Breeze Nova Diffusers were positioned symmetrically in the two corners of the experimental room. For each session, 10 drops (approximately 0.5 mL) of undiluted essential oil were applied to the internal absorbent cotton pad of each diffuser. All devices were activated 10 minutes prior to participant entry to allow stable odor dispersion throughout the room and remained active during the entire experimental session to maintain continuous ambient exposure. The diffusers were positioned outside participants’ direct line of sight to minimize visual awareness of the odor source. Participants were not instructed to inhale directly from any device

2. Specification of image color normalization procedure

Comment:
Please clarify the description of “global color normalization.”

Response:
We appreciate this request for greater methodological precision. In the previous version, the term “global color normalization” was used imprecisely. In the revised manuscript, we clarify that preprocessing consisted only of standardized RGB conversion and resizing to a fixed resolution (200 × 200 pixels).

Specifically:

- Images were loaded using the Python Imaging Library (PIL).
- They were converted to 8-bit RGB color space using the .convert("RGB") function.
- Images were resized to 200 × 200 pixels using PIL’s default resampling parameters.

No histogram equalization, channel scaling, intensity rescaling, or post hoc white-balance correction was applied.

Because all paintings were photographed under controlled indoor lighting with fixed camera parameters (ISO, aperture, and white balance), additional computational normalization procedures were considered unnecessary and were intentionally avoided to preserve original pixel-value distributions for k-means color clustering.

We have revised the Analysis section accordingly. The complete preprocessing and clustering pipeline is publicly available on OSF (<https://osf.io/cnre7/>).

Lines 216-235, 521-522:

Python and R were used for statistical analyses. Image preprocessing and color extraction were conducted in Python. Images were loaded using the Python Imaging Library (PIL), converted to 8-bit RGB color space using the .convert("RGB") function, and resized to a standardized resolution of 200 × 200 pixels using PIL’s default resampling parameters to ensure uniform spatial sampling across images.

Pixel values were reshaped into RGB triplets and submitted to k-means clustering (k = 25; random_state = 0; n_init = 10) to estimate dominant color centroids for each painting. Images were acquired under controlled indoor lighting conditions with fixed camera parameters (ISO, aperture, and white balance), as well as constant camera-to-painting distance and background. No additional histogram equalization, channel scaling, intensity normalization, or post hoc white-balance correction was applied, as acquisition conditions were standardized to minimize illumination variability while preserving original pixel-value distributions for clustering.

The number of clusters was fixed at 25 for all paintings, corresponding to a 5 × 5 color palette. This selection reflects a balance between capturing fine-grained color variation and maintaining comparability across images, consistent with established approaches in digital color analysis and computational aesthetics [24].

3. Minor language and typographic polishing

Comment:
Please ensure consistency in capitalization, hyphenation, spacing, and statistical notation.

Response:
We have conducted a thorough proofreading of the entire manuscript. Specifically:

- Hyphenation has been standardized (e.g., cross-modal, odor-color, perceptual-sensory, affective-evaluative).
- En dashes used within compound terms were replaced with standard hyphens.
- Statistical notation was harmonized (e.g., p < .001, F(1, 47.15), partial η²).
- Spacing inconsistencies and minor typographic issues were corrected.
- Capitalization of section headings and factor labels was standardized.

These revisions improve stylistic consistency and clarity throughout the manuscript.

Comment:
Some statements may be slightly over-assertive.

Response:
We carefully reviewed the Abstract and Discussion and adjusted wording where appropriate to ensure balanced interpretation. Overly strong terms were replaced with more cautious phrasing (e.g., “confirm” replaced with “provide evidence for” or “suggest”), and conclusions were refined to reflect the scope and limitations of the data.

We are grateful for the editor’s constructive comments, which have helped us improve the clarity, transparency, and methodological precision of the manuscript. We believe the revised version addresses all remaining concerns and enhances the overall rigor and reproducibility of the work.

With sincere thanks,
Nobuyuki Sakai,
(on behalf of all authors)
